# Novel pegylated silver coated carbon nanotubes kill *Salmonella* but they are non-toxic to eukaryotic cells

**DOI:** 10.1186/s12951-015-0085-5

**Published:** 2015-03-22

**Authors:** Atul A Chaudhari, Shanese L Jasper, Ejovwoke Dosunmu, Michael E Miller, Robert D Arnold, Shree R Singh, Shreekumar Pillai

**Affiliations:** Center for Nanobiotechnology Research, Alabama State University, Montgomery, AL USA; Research Instrumentation Facility, Auburn University, Auburn, AL USA; Department of Drug Discovery and Development, Auburn University, Auburn, AL USA

**Keywords:** Pegylation, Silver coated carbon nanotubes, Toxicity, Gene regulation, Food borne

## Abstract

**Background:**

Resistance of food borne pathogens such as *Salmonella* to existing antibiotics is of grave concern. Silver coated single walled carbon nanotubes (SWCNTs-Ag) have broad-spectrum antibacterial activity and may be a good treatment alternative. However, toxicity to human cells due to their physico-chemical properties is a serious public health concern. Although pegylation is commonly used to reduce metal nanoparticle toxicity, SWCNTs-Ag have not been pegylated as yet, and the effect of pegylation of SWCNTs-Ag on their anti-bacterial activity and cell cytotoxicity remains to be studied. Further, there are no molecular studies on the anti-bacterial mechanism of SWCNTs-Ag or their functionalized nanocomposites.

**Materials and methods:**

In this study we created novel pegylated SWCNTS-Ag (pSWCNTs-Ag), and employed 3 eukaryotic cell lines to evaluate their cytotoxicity as compared to plain SWCNTS-Ag. Simultaneously, we evaluated their antibacterial activity on *Salmonella enterica* serovar Typhimurium (*Salmonella* Typhimurium) by the MIC and growth curve assays. In order to understand the possible mechanisms of action of both SWCNTs-Ag and pSWCNTs-Ag, we used electron microscopy (EM) and molecular studies (qRT-PCR).

**Results:**

pSWCNTs-Ag inhibited *Salmonella* Typhimurium at 62.5 μg/mL, while remaining non-toxic to human cells. By comparison, plain SWCNTs-Ag were toxic to human cells at 62.5 μg/mL. EM analysis revealed that bacteria internalized either of these nanocomposites after the outer cell membranes were damaged, resulting in cell lysis or expulsion of cytoplasmic contents, leaving empty ghosts. The expression of genes regulating the membrane associated metabolic transporter system (*artP*, *dppA*, and *livJ*), amino acid biosynthesis (*trp* and *argC*) and outer membrane integrity (*ompF*) protiens, was significantly down regulated in *Salmonella* treated with both pSWCNTs-Ag and SWCNTs-Ag. Although EM analysis of bacteria treated with either SWCNTs-Ag or pSWCNTs-Ag revealed relatively similar morphological changes, the expression of genes regulating the normal physiological processes of bacteria (*ybeF*), quorum sensing (*sdiA*), outer membrane structure (*safC*), invasion (*ychP*) and virulence (*safC*, *ychP*, *sseA* and *sseG*) were exclusively down regulated several fold in pSWCNTs-Ag treated bacteria.

**Conclusions:**

Altogether, the present data shows that our novel pSWCNTs-Ag are non-toxic to human cells at their bactericidal concentration, as compared to plain SWCNTS-Ag. Therefore, pSWCNTs-Ag may be safe alternative antimicrobials to treat foodborne pathogens.

**Electronic supplementary material:**

The online version of this article (doi:10.1186/s12951-015-0085-5) contains supplementary material, which is available to authorized users.

## Background

Antibiotic resistance of foodborne pathogens is a matter of serious concern as it has a detrimental effect not only on human health, but also results in food deterioration thus requiring immediate attention [1]. The use of nanoparticles appears to be one of the most promising strategies for overcoming microbial resistance as development of resistance to these nanoparticles is not likely [2]. Antimicrobial nanoparticles (NP) have been reported to be effective antibacterial agents due to their high surface area to volume ratios, unique physicochemical properties and multiple mechanisms of antimicrobial action that prevent development of microbial resistance to these nanoparticles [3,4]. Of relevance, silver nanoparticles (AgNPs) have garnered attention due to their ability to kill both gram-positive and gram-negative bacteria [5-8]. However, due to their high surface energy, AgNPs tend to aggregate into large particles which may affect their bactericidal property due to instability in the growth medium [9]. Alternatively, the use of carbon nanotubes (CNTs) as a host nanomaterial [10] for AgNPs creates a stable nanocomposite [8,11-13]. CNTs have been shown to be a suitable vehicle for the efficacious and target specific delivery of molecules [10,14]. Silver coated CNTs (AgCNTs) have demonstrated stronger antibacterial activity against gram-positive as well as gram-negative bacteria compared to commercially available AgNPs [8,13].

However, AgNPs or CNTs are toxic to human cells, with several known mechanisms of toxicity to eukaryotic cells [2,15-21]. Toxicity of AgNPs or CNTs can be reduced using various functionalization strategies, which also improves their solubility and dispersability [20-26]. More recently, neutral amphiphiles of single walled carbon nanotubes have been shown to be less toxic and stable in various media [10,14]. Specifically, pegylation of CNTs using polyethylene glycol (PEG) has been shown to increase biocompatibility and reduce the toxicity of CNTs administered intravenously in mice as compared to non-pegylated CNTs [24]. As yet, most research has focused on how the toxicity of either plain AgNPs or CNTs could be reduced by pegylation. However, AgCNTs have not been pegylated as yet, and the effect of pegylation on their toxicity to human cell has not been investigated. Further, it also remains to be determined whether pegylation of AgCNTs affect their antibacterial activity, as PEG molecules may cover the silver coating on CNTs, thus reducing their antibacterial activity. Another gap in this area is that very little is known about the antibacterial mechanism of AgCNTs and pegylated AgCNTs. Some of the proposed mechanisms include silver ion dissolution, direct contact with cell membranes and generation of reactive oxygen species [9]. A recent study demonstrated that the antibacterial activity of AgCNTs is mediated through generation of reactive oxygen species via its direct contact with the bacterial cells [9]. However, the molecular mechanisms of action remain to be explored.

Accordingly, in the present study we created novel pegylated silver coated single walled carbon nanotubes (pSWCNTs-Ag) using PEG and employed 3 eukaryotic cell lines to evaluate their cytotoxicity as compared to plain SWCNTS-Ag. Simultaneously, we evaluated their antibacterial activity on *Salmonella* Typhimurium, a gram-negative foodborne pathogen of serious public health concern, using the MIC and growth curve assays. Further, to understand the possible mechanisms of action of both SWCNTs-Ag and pSWCNTs-Ag, we performed electron microscopy (EM) and molecular studies using quantitative reverse transcriptase polymerase chain reaction (qRT-PCR).

## Results

### Characterization of pSWCNTs-Ag and SWCNTs-Ag

**Figure 1:**
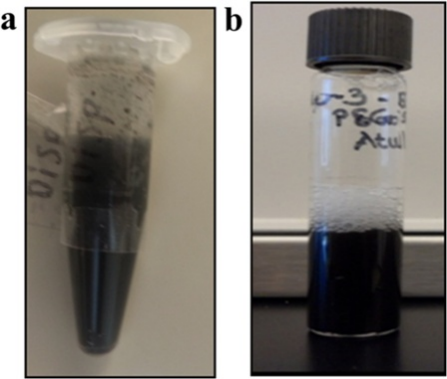
**Suspensions of silver coated single walled carbon nanotubes (SWCNTs-Ag). (a)** Homogenously dispersed SWCNTs-Ag in NanoSperse AQ®. **(b)** Water soluble pSWCNTs-Ag.

**Figure 2:**
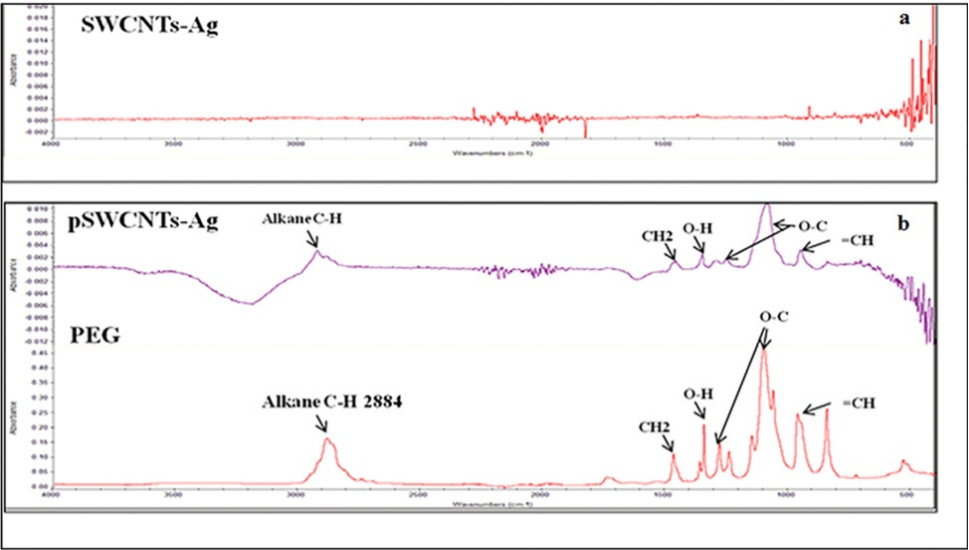
**FT-IR pattern of (a) SWCNTs-Ag. (b) pSWCNTs-Ag and PL-PEG.** The characteristic peaks on PL-PEG and pSWCNTs-Ag such as alkane C-H, carbonyl c = o, hydroxyl O-H and methylene CH_2_ are indicated by arrows.

**Figure 3:**
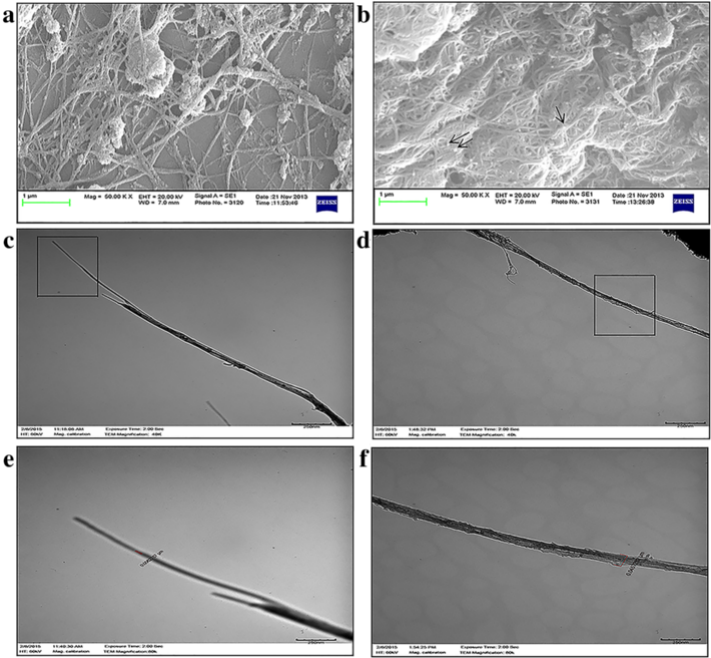
**Characterization of silver coated single walled carbon nanotube formulations by SEM (a & b) and TEM (c & d). (a)** SEM image of silver coated single walled carbon nanotubes dispersed in NanoSperse AQ® dispersant (SWCNTs-Ag). **(b)** SEM image of pegylated SWCNTs (pSWCNTs-Ag). The hazy coating around the SWCNTs-Ag is apparent and silver deposition is indicated by an arrow. **(c)** TEM image of SWCNTs-Ag. **(d)** TEM of pSWCNTs-Ag. **(e)** Magnified inset of image C showing the diameter of SWCNT-Ag as 6 nm. **(f)** Magnified inset of image D showing pSWCNTs-Ag of 54 nm.

As indicated by the manufacturer, dispersion of SWCNTs-Ag in NanoSperse AQ® resulted in a relatively homogenous, yet insoluble suspension, whereas pegylation of SWCNTs-Ag produced a homogenous and highly stable water soluble suspension as reported elsewhere (Figure 1) [27]. The zeta potential value of pSWCNTs-Ag (Table 1) was positive (+8.99) compared to the negatively charged SWCNTs-Ag (−41.9), indicating that phospholipid-poly (ethylene glycol)**-**amine (PL-PEG-amine) molecules were strongly anchored over SWCNTs-Ag, which imparted the positive charge and made them water-soluble. Fourier transform infrared spectroscopy (FT-IR) analysis showed the presence of characteristic peaks of PL-PEG (alkane C-H, carbonyl c = o etc.) on pSWCNTs-Ag, whereas SWCNTs-Ag did not possess any similar peaks (Figure 2). Further, SEM imaging of pSWCNTs-Ag clearly indicated a cloudy hazy coating of PEG around the SWCNTs-Ag (Figure 3b, silver can be seen as spherical deposits, indicated by arrowheads) as compared to SWCNTs-Ag with no coating around them (Figure 3a). TEM images clearly verified the pegylation as evidenced by increase in size of pSWCNTs-Ag (54 nm) (Figure 3d & f) compared to SWCNTs-Ag (6 nm) (Figure 3c & e). The amount of PL-PEG that was deposited on 10 mg/mL of SWCNTs-Ag was observed to be 2 μM equivalent to 10 mg/mL as measured by the inorganic phosphate assay.

**Table 1:** **Zeta potential measurements of the nanocomposites**

**Nanocomposite**	**Zeta potential (mV)**	**STDEV**
SWCNTs-Ag	−41.9	1.30
pSWCNTs-Ag	8.99	0.52

### Antibacterial activity of SWCNTs-Ag

**Figure 4:**
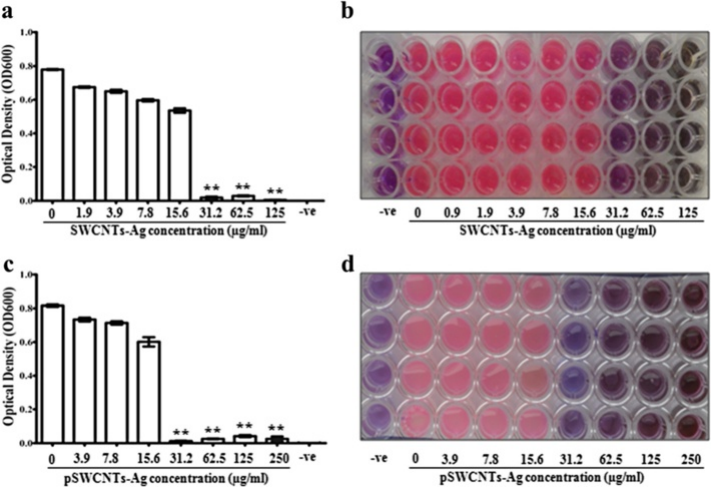
**Evaluation of the minimum inhibitory concentrations (MICs) using the redox resazurin dye-based microtiter broth dilution assay.** 1 × 10^5^ cfu/mL bacteria were exposed to doubling concentrations of nanocomposites. **(a)** SWCNTs-Ag without resazurin. **(b)** SWCNTs-Ag with resazurin. **(c)** pSWCNTs-Ag without resazurin. **(d)** pSWCNTs-Ag with resazurin. All the plates were incubated at 37°C and the optical density at 600 nm (OD600) was determined after 24 h. All values were considered to be significant at p ≤ 0.05 or 0.01 versus the controls (0 μg/mL of SWCNTs-Ag present in bacterial culture). **p ≤ 0.01 indicating highly significant differences. Error bars represent standard deviations determined from at least six replicates.

**Figure 5:**
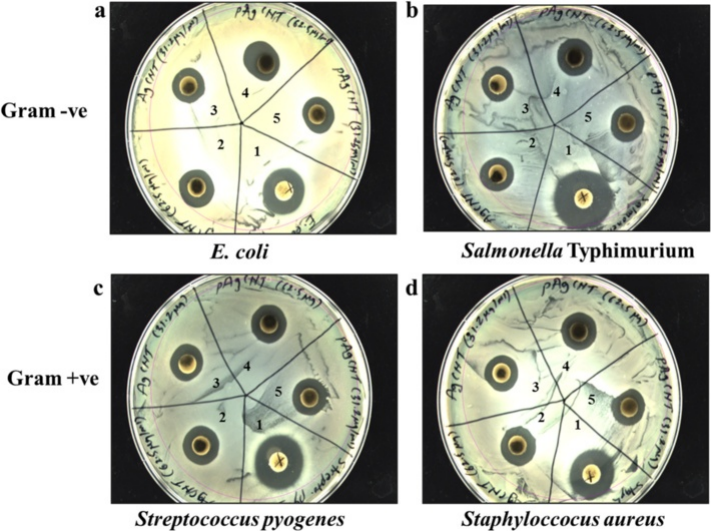
**Zone of inhibition test using Kirby–Bauer disc diffusion assay against gram- negative and gram-positive organisms.** The zone of inhibition around discs containing MIC concentrations of SWCNTs-Ag and pSWCNTs-Ag and the broad spectrum antibiotic amoxicillin–clavulanic acid (30 μg) can be clearly seen for both, the gram- negative pathogens such as **(a)** *Escherichia coli* and **(b)** *Salmonella* Typhimurium; and gram-positive pathogens such as **(c)** *Streptococcus pyogenes* and **(d)** *Staphylococcus aureus*. The numbers indicated are; 1: antibiotic control; 2: SWCNTs-Ag (62.5 μg/mL); 3: SWCNTs-Ag (31.25 μg/mL); 4: pSWCNTs-Ag (62.5 μg/mL); 5: pSWCNTs-Ag (31.25 μg/mL).

**Figure 6:**
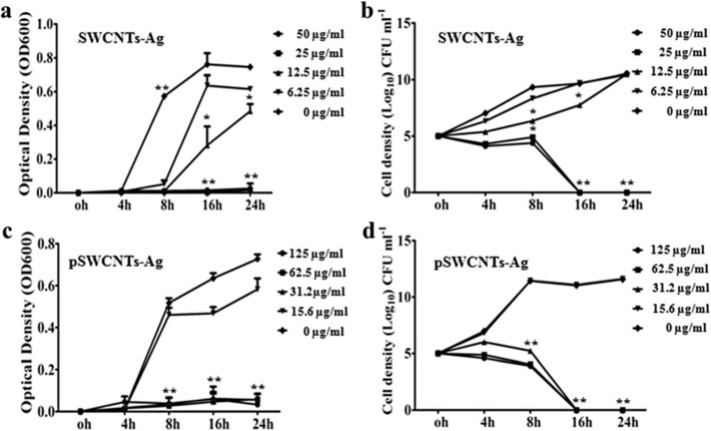
**Growth curve and quantitative analysis of** ***Salmonella*** **Typhimurium exposed to various concentrations of nanocomposites. (a)** Bacterial growth curve exposed to SWCNTs-Ag using optical density measurements. **(b)** cfu/mL of surviving bacteria upon exposure to SWCNTs-Ag. **(c)** Growth curve upon exposure to pSWCNTs-Ag using optical density measurements. **(d)** Quantification of bacteria upon exposure to pSWCNTs-Ag. Bacteria were grown in LB broth containing various concentrations of nanocomposites and all the cultures were incubated at 37°C with shaking at 250 rpm and the optical density measurements at 600 nm (OD600) and cfu/mL counts were done at 0, 4, 8, 16 and 24 h. The results are means of three experiments, with p ≤ 0.05 indicating significant * differences, or p ≤ 0.01 indicating highly significant ** differences. Error bars represent standard deviations determined from at least three replicates.

**Figure 7:**
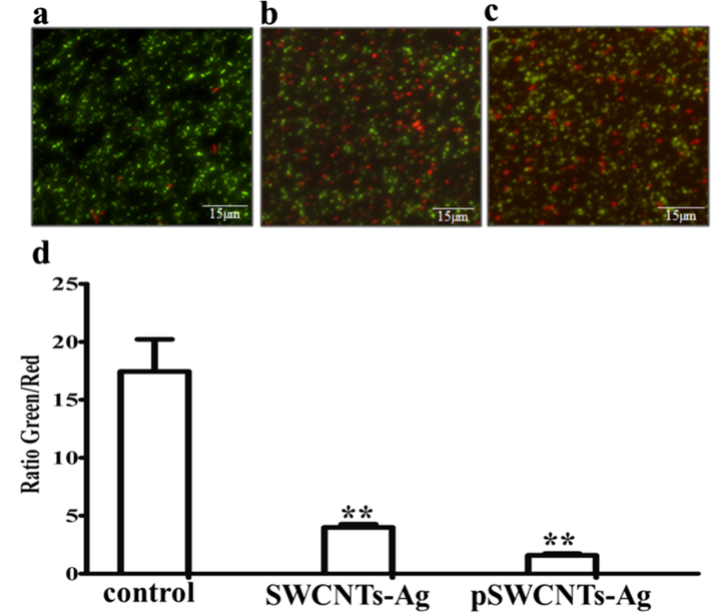
**Live/ dead staining of bacteria exposed to different nanocomposites.** 1 × 10^5^ cfu/mL bacteria were exposed to 12.5 μg/mL of nanocomposites for 16 h and bacteria were stained using Baclight bacterial viability kits. **(a)** Non-treated bacteria. **(b)** Bacteria exposed to SWCNTs-Ag **(c)** pSWCNTs-Ag treated bacteria. **(d)** Ratio of green/red colored bacteria upon exposure to nanocomposites. All values were considered to be significant * at p ≤ 0.05 * or ** highly significant at p ≤ 0.01. Error bars represent standard deviations of the results determined with at least six replicates. The images are at 10× magnification. Scale bars: ~15 μm

Next, we thoroughly examined the antibacterial activity of SWCNTs-Ag and pSWCNTs-Ag. The MIC values for both, SWCNTs-Ag and pWCNTs-Ag, were between 31.25 μg/mL and 62 μg/mL for *Salmonella* Typhimurium, *Escherichia coli*, *Staphylococcus aureus* and *Streptococcus pyogenes* (Figure 4; Additional file 1: Figures S1-S3). Additionally, the Kirby Bauer disc diffusion assay demonstrated strong antibacterial activity against all four pathogens characterized by zones of inhibition on the MH agar plates (Figure 5; Additional file 1: Table S1). Further, the growth curve assay of *Salmonella* Typhimurium exposed to various concentrations of SWCNTs-Ag showed that bacterial growth was dramatically inhibited in a time and concentration dependent manner (Figure 6). Similarly, pSWCNTs-Ag exhibited strong antibacterial activity at 125, 62.5 and 31.25 μg/mL as evidenced by reduced bacterial numbers and impeded growth as the time progressed (Figure 6). Based on the bacterial growth curve analysis, we further analyzed live/dead staining of bacteria upon exposure to 12.5 μg/mL of SWCNTs-Ag and pSWCNTs-Ag for 16 h. The live/dead proportion was approximately 7–8 fold lower when bacteria were exposed to SWCNTs-Ag and pSWCNTs-Ag compared to non-treated controls (Figure 7).

### Pegylated SWCNTs-Ag are non-toxic to human cells at their bactericidal concentrations

**Figure 8:**
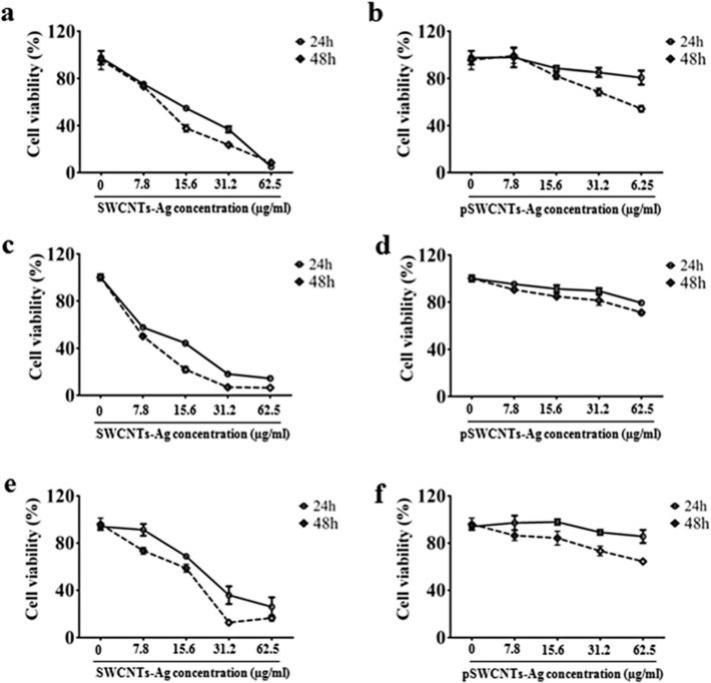
***In vitro*** **cytotoxicity assay for SWCNTs-Ag and pSWCNTs-Ag. (a)** Viability of A549 cells exposed to SWCNTs-Ag. **(b)** Cell viability of A549 cells exposed to various concentrations of pSWCNTs-Ag. **(c)** J774 cell viability exposed to SWCNTs-Ag. **(d)** Cell viability of J774 treated with pSWCNTs-Ag. **(e)** Hep-2 cells viability treated with SWCNTsAg. **(f)** Viability of Hep-2 cells exposed to pSWCNTs-Ag. All the cells were treated with nanocomposites for 24 and 48 h and statistical differences were calculated compared to controls. All values were expressed as fold change expressed compared to non-treated bacteria. ^*^when *p* ≤ 0.05 indicate significant differences; ^**^when *p* ≤ 0.01 indicate highly significant differences. Error bars represent standard deviations of the results determined with at least 3 biological replicates.

**Figure 9:**
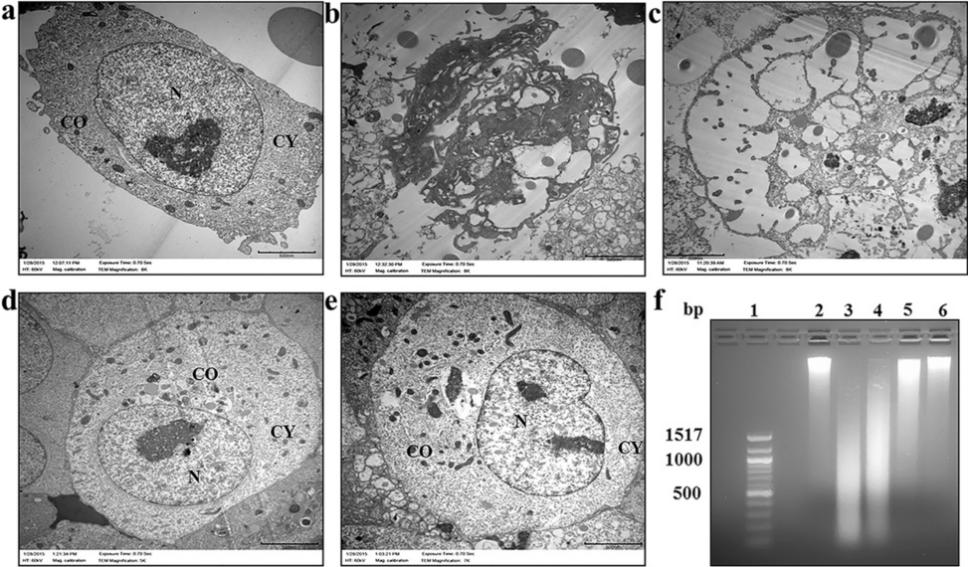
**Toxicity evaluation of SWCNTs-Ag and pSWCNTs-Ag in A549 cells by TEM (a-e) and DNA fragmentation assay (f). (a)** non-treated A549 cells. **(b)** A549 cells treated with 31.25 μg/mL of SWCNTs-Ag. **(c)** A549 cells treated with 15.6 μg/mL of SWCNTs-Ag. **(d)** A549 cells treated with 31.25 μg/mL of pSWCNTs-Ag. **(e)** A549 cells treated with 15.6 μg/mL of pSWCNTs-Ag, N: nucleus; CY: cytoplasm; CO: cell organelle. **(f)** DNA fragmentation assay, Lane 1: 100 bp DNA ladder; 2: non treated control; 3: cells treated with 31.25 μg/mL of SWCNTs-Ag; 4: cells treated with 15.6 μg/mL of SWCNTs-Ag; 5: cells treated with 31.25 μg/mL of pSWCNTs-Ag; 6: cells treated with 15.6 μg/mL of pSWCNTs-Ag.

The cytotoxicity of SWCNTs-Ag and pSWCNTs-Ag was compared by the MTT assay using 3 cell lines including the A549 (human lung carcinoma), Hep2 (hepatocellular carcinoma) and J774 (murine macrophages) cells. As represented in Figure 8, SWCNTs-Ag showed dose dependent toxicity in all the cell lines, whereas pSWCNTs-Ag were observed not to be toxic at similar concentrations. Further, A549 cells treated with plain SWCNTs-Ag (32.25 & 15.6 μg/mL) showed damaged morphology such as loss of nucleus, cell organelles and presence of apoptotic vesicles upon TEM analysis (Figure 9b & c). On the other hand, cells exposed to pSWCNTs-Ag at similar concentrations as that of SWCNTs-Ag showed normal morphology including intact nuclei and cell organelles (Figure 9d & e) similar to non-treated control cells (Figure 9a). Additionally, the DNA fragmentation assay showed fragmented DNA, a characteristic of apoptotic cell death, when treated with SWCNTs-Ag whereas pSWCNT-Ag treated cells did not show DNA fragmentation similar to the non-treated control cells (Figure 9f).

### EM analysis of *Salmonella* Typhimurium exposed to SWCNTs-Ag and pSWCNTs-Ag

**Figure 10:**
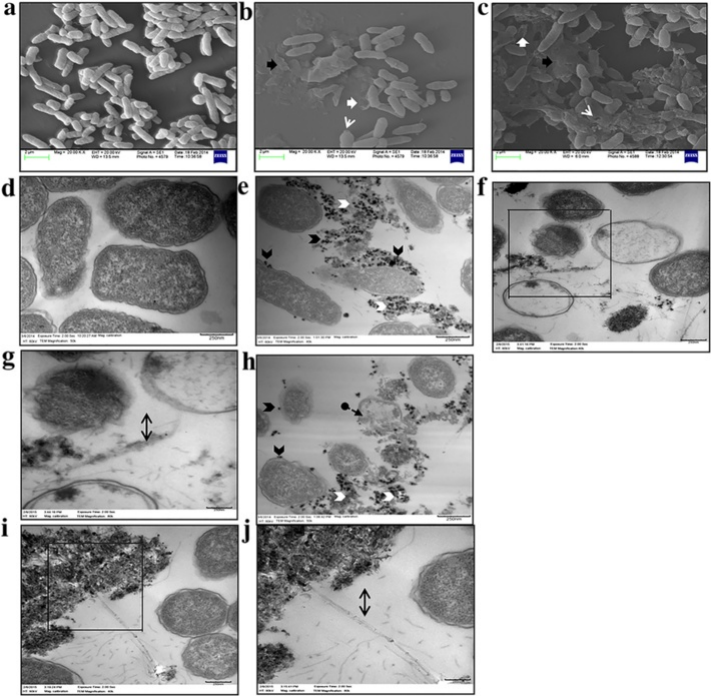
**Evaluation of morphological changes in bacteria upon their interaction with SWCNTs-Ag and pSWCNTs-Ag by SEM (a-c) and TEM (d-j). (a)** Non treated *Salmonella* Typhimurium. **(b)** *Salmonella* Typhimurium treated with SWCNTs-Ag. **(c)** Treatment with pSWCNTs-Ag. **(d)** Non-treated bacteria. **(e-g)** Bacteria exposed to SWCNTs-Ag; **(g)** is magnified inset of **(f). (h-j)** Bacteria exposed to pSWCNTs-Ag; **(j)** is magnified inset of **(i)**. Black solid arrows indicate lysis of the cells. White solid block arrows indicate dissolved entities. White line arrows indicate pore formation. Black arrow-heads represent nanocomposites, whereas white arrow-heads indicate cell debris. Dotted line black arrows show empty ghost cells. Two headed arrow indicates the presence of nanocomposites.

The SEM and TEM images of either healthy non-treated bacterial cells or nanocomposites-treated cells are shown in Figure 10. SEM analysis revealed that treatment with the SWCNTs-Ag or pSWCNTs-Ag resulted in the complete lysis of the bacterial cells (indicated by a black solid arrow), or the cells appeared as hollow disrupted entities (indicated by a white solid block arrow) compared to non-treated healthy bacterial cells (Figure 10a). Some of the bacterial cells also showed the presence of holes in either a central division region (Figure 10b), or a polar division region (Figure 10c indicated by white line arrows). These findings were further supported by TEM imaging (Figure 10d-j), which showed damaged bacterial cells upon treatment with the nanocomposites (Figure 10e & j) compared to healthy untreated bacteria (Figure 10d). Upon interaction with the nanocomposites (indicated by double headed black arrows), bacterial cell membranes were ruptured causing the expulsion of cytoplasmic contents outside the cells, leaving behind either an empty ghost cell (Figure 10h, indicated by a dotted line black arrow) or debris of the cell (indicated by a white arrowhead). By comparison, non-treated bacteria had intact cell membranes and cytoplasmic contents (Figure 10d).

### Molecular studies of *Salmonella* Typhimurium exposed to nanocomposites

**Figure 11:**
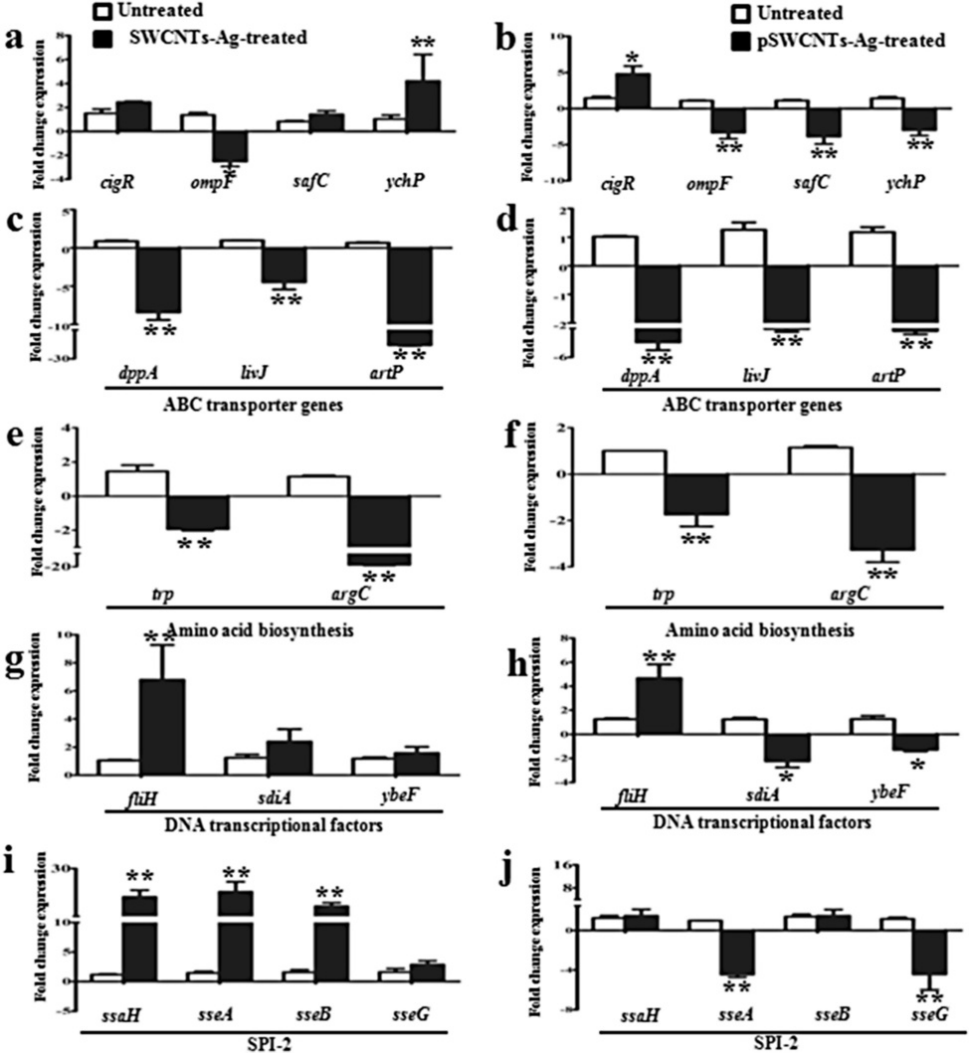
**Gene expression studies in** ***Salmonella*** **Typhimurium exposed to either SWCNTs-Ag or pSWCNTs-Ag nanocomposites. (a)** and **(b)** represent gene expression of *cigR*, *ompF*, *safC* and *ychP* upon exposure to SWCNTs-Ag and pSWCNTs-Ag, respectively. **(c)** and **(d)** represent ABC transporter systems-associated gene expression in bacteria treated with SWCNTs-Ag and pSWCNTs-Ag, respectively. **(e)** and **(f)** represent the expression of amino acid biosynthesis genes in bacteria exposed to SWCNTs-Ag and pSWCNTs-Ag, respectively. **(g)** and **(h)** represent expression of genes related to DNA transcription in bacteria treated with SWCNTs-Ag and pSWCNTs-Ag, respectively. **(i)** and **(j)** indicate the expression of genes associated with the SPI-2 type three secretion system in bacteria exposed to SWCNTs-Ag and pSWCNTs-Ag, respectively. All values were expressed as fold change expression compared to non-treated bacteria. ^*^when *p* ≤ 0.05 indicate significant differences; ^**^when *p* ≤ 0.01 indicate highly significant differences. Error bars represent standard deviations of the results determined with at least 3 biological replicates.

The expression of genes associated with bacterial metabolism, structural integrity and virulence was investigated to further explore the antibacterial action of nanocomposites. Bacteria treated with plain SWCNTs-Ag showed down-regulation of the *ompF* gene and up-regulation of the *ychP* gene whereas there was no significant change in expression of the genes such as *cigR* and *safC* (Figure 11). On the other hand, pSWCNTs-Ag treated cells showed significant down regulation of *ompF*, *safC* and *ychP* genes whereas the *cigR* gene was up regulated (Figure 11). Further, the genes such as *dppA*, *livJ*, *artP, trpA* and *argC* were significantly down-regulated several-fold upon exposure to SWCNTs-Ag and pSWCNTs-Ag (Figure 11c-f) whereas the expression of the *fliH* gene was significantly up-regulated upon treatment with both the nanocomposites (Figure 11g & h). The expression of *ybeF* and *sdiA* was down-regulated significantly in pSWCNTs-Ag treated bacteria, whereas the expression of these two genes remained unchanged upon SWCNTs-Ag treatment (Figure 11g & h). Also, the bacteria treated with plain SWCNTs-Ag showed significant up regulation of genes such as *sseA sseB* and *ssaH* whereas the expression of gene *sseG* remained unchanged (Figure 11i). Conversely, pSWCNTs-Ag treated bacteria showed down regulation of *sseA* and *sseG* genes with no change in expression of *sseB* and *ssaH* genes (Figure 11h).

## Discussion

Resistance of food borne pathogens to antibiotics is a serious problem and the development of resistance-free antibacterial agents is necessary to treat bacterial infections effectively. Although the use of SWCNTs-Ag is gaining popularity due to their excellent antibacterial properties, toxicity to human cells remains a major concern. Results of our study show that pegylation of SWCNTs-Ag reduces their toxicity to eukaryotic cells, compared to plain non-pegylated SWCNTs-Ag, and our results are in agreement with the previous findings that show pegylation reduces the toxicity of plain SWCNTs [22,28]. Additionally, our findings also corroborate with the recent findings wherein biotinylated amphiphiles of SWCNTs have been shown to reduce the toxicity to various mammalian cell lines [10,14]. However, to our knowledge, this is the first attempt to pegylate SWCNTs-Ag and to report that the pSWCNTs-Ag were non-toxic to human cells. Further, the effect of pegylation on the antibacterial activity of SWCNTs-Ag also remains to be reported. As the SWCNTs used in the present study were silver coated, it is possible that the anti-bacterial activity of SWCNTs-Ag could have been compromised by pegylation, as PL-PEG-amine molecules may have covered the silver coating on the SWCNTs. However, our results clearly demonstrated that pegylated SWCNTs-Ag destroy *Salmonella* as effectively as plain SWCNTs-Ag. The bacterial MIC values for pSWCNTs-Ag did not differ from that of plain SWCNTs-Ag (62.5 & 31.2 μg/mL). Sequential monitoring of quantitative bacterial growth over time, and live/dead staining of bacteria upon exposure to various concentrations of nanocomposites provided further evidence that the antibacterial activity of SWCNTs-Ag was not reduced by pegylation. At 31.2 μg/mL concentration, both the nanocomposites exhibited bactericidal activity, however, the uncoated SWCNTs-Ag were cytotoxic. By contrast, pSWCNTs-Ag were non-toxic to all the cell lines at bactericidal concentrations of 62.5 μg/mL, which is at least twice the MIC value. The higher toxicity of plain non-pegylated SWCNTs-Ag may be attributed to their lower surface area as a result of their tendency to agglomerate and their surface chemistry [17]. Due to their insolubility and agglomerative properties, SWCNTs-Ag may not be able to enter cells thus inducing contact mediated cell toxicity similar to CNTs [22]. On the contrary, pSWCNTs-Ag were soluble in water and therefore showed reduced toxicity, or no toxicity, possibly due to their appropriate distribution in the solution, lessening the contact mediated toxicity due to their ability to enter in to human cells similar to pegylated CNTs [22].

Next, we also investigated the morphological changes seen in *Salmonella* upon exposure to either plain or pegylated SWCNTs-Ag using electron microscopy. SEM and TEM clearly showed that nanocomposites damaged the bacterial cells by either creating a hole on the bacterial cell surface, or causing lysis of the bacterial cells. This mechanism of action results from the Ag coating of SWCNTs, as AgNPs are well known for contact-mediated membrane damage [5,6,29]. Further, SWCNTs by themselves have been shown to cause membrane damage in bacteria thereby regulating their antibacterial activity [30,31]. However, all these studies have shown the membrane damaging effects of AgNPs or CNTs by SEM analysis and lack detailed information about further validation by TEM. More recently, the antibacterial activity of Ag-doped multi-walled CNTs has been associated with the generation of reactive oxygen species through direct contact of the nanocomposites with bacteria causing membrane damage [9]. Our study indicated additional possible mechanisms for the antibacterial activity of SWCNTs-Ag, perhaps linked with the well-known ghost formation phenomenon, as characterized by tunnel formation on bacterial cell membranes, resulting in the expulsion of the cytoplasmic contents [32]. However this may require further validation by more advanced analyses using atomic force microscopy [33] or in- lens field emission scanning electron microscopy [34] to capture the detailed morphological changes induced by SWCNTs-Ag in a sequential manner.

As EM studies revealed that SWCNTs-Ag caused membrane damage and formation of ghost cells, we further investigated the expression of a few, yet major, genes associated with invasion (*ychP*), outer membrane proteins (*ompF* and *safC*), inner membrane protein (*cigR*), amino acid biosynthesis (*trpA*, tryptophan and a*rgC*, arginine), ABC transport (*dppA*, *artP* and *livJ*), DNA transcriptionactivators (*fliH*, *sdiA* and *ybeF*) and *Salmonella* pathogenicity island-2 (SPI-2) associated genes (*sseA*, *sseB*, *sseG* and *ssaH*) using qRT-PCR [35,36]. These genes were selected either on the basis of their common existence and physiological roles in a wide range of bacterial species, or their significant role in *Salmonella* virulence. The expression of genes associated with membrane integrity (*ompF*, *cigR*, *ychP*) was significantly down regulated by treatment with plain silver or pegylated silver nanocomposites. However, another outer membrane associated gene *safC* (associated with SPI-6) and the *ychP* invasin gene were exclusively down regulated only in pSWCNTs-Ag treated cells. These results support our hypothesis that both the nanocomposites cause bacterial cell membrane damage, as evidenced by change in gene expression that regulates proteins associated with the bacterial membranes. Our results also indicate that pSWCNTs-Ag interfere with the expression of additional genes compared to plain SWCNTs-Ag, which can be attributed to the solubility of pSWCNTs-Ag in water. Further, the genes regulating the bacterial membrane associated ABC transporter proteins and amino acid synthesis were down regulated significantly by either nanocomposites treatment. ABC transporters are extremely vital in cell viability, virulence and pathogenicity as they function as protein systems that counteract any undesirable changes occurring in the cell [37]. The periplasmic DppA [38] (initial receptor for dipeptides transport), artP [39] (an important component of the arginine binding and transport system) and livJ [40] (regulator of three branched-chain amino acids system) proteins are some of the major ABC transporters that allow transport of a wide variety of materials across cytoplasmic membranes, and play an important role in bacterial metabolism under conditions of stress. Down regulation of the genes regulating these proteins indicates the inhibition of these bacterial recovery mechanisms. Similarly, the down- regulation of tryptophan (an essential amino acid) and arginine biosynthesis genes further suggests that Ag nanocomposites may also interfere with the amino acid synthesis machinery of bacteria.

On the other hand, the *fliH* gene, which encodes the flagellar FliH protein was significantly up-regulated upon exposure to both nanocomposites. FliH is an effector protein involved in the flagellar export apparatus which regulates the transcription of *flaB* and thus the motility of bacteria [41]. Up-regulation of *fliH* in treated bacteria thus, may indicate damage to the flagellar assembly, and a stress recovery response from the damage caused by the Ag nanocomposites.

In addition to these molecular changes, only the bacteria treated with pegylated SWCNTs-Ag showed differential expression of the genes associated with biofilm formation, quorum sensing and virulence. The expression of *ybeF* (a transcriptional regulator of the lysR family) and *sdiA* (a receptor for N-acyl-L-homoserine lactones), was down regulated significantly in pSWCNTs-Ag treated bacteria, whereas the expression of these two genes remained unchanged upon SWCNTs-Ag treatment (Figure 11g & h). The lysR family of protein regulators play a diverse role in cellular functions such as transport, response to oxidative stress, nitrogen fixation, biofilm formation and bacterial virulence, whereas sdiA proteins play a key role in quorum sensing mechanisms of pathogenic bacteria [42,43]. Our results thus indicate that pSWCNTs-Ag may have an additional capability of interfering with normal physiological processes and quorum sensing mechanisms of bacteria compared to non-pegylated plain SWCNTs-Ag. Similarly, the genes associated with the SPI-2 mediated type three-secretion system (T3SS) showed different expression patterns upon exposure to either nanocomposite. The expression of *sseA* was significantly down regulated in bacteria treated with pSWCNTs-Ag, which resulted in unchanged expression of the *sseB* and *ssaH* genes, and down regulation of the *sseG* gene. Conversely, the expression of *sseA* was up-regulated, followed by several fold increase in *sseB* and *ssaH* expression when the bacteria were treated with SWCNTs-Ag. SPI-2 T3SS is required for intracellular survival of *Salmonella*, and development of systemic disease [44]. The *sseA* gene of SPI-2 T3SS plays an important role in pathogenesis and acts as a chaperone to regulate the stabilization and export of the *sseB* filament protein and other effector proteins such as *sseG* and *ssaH* [45].Thus, our findings indicate that pSWCNTs-Ag may affect several pathways to exert their antibacterial effect, as compared to plain SWCNTs-Ag. Further studies are required to fully elucidate the antibacterial effect of both nanocomposites on the bacterial genome using more advanced methods such as DNA microarray or whole genome sequencing.

## Conclusions

**Figure 12:**
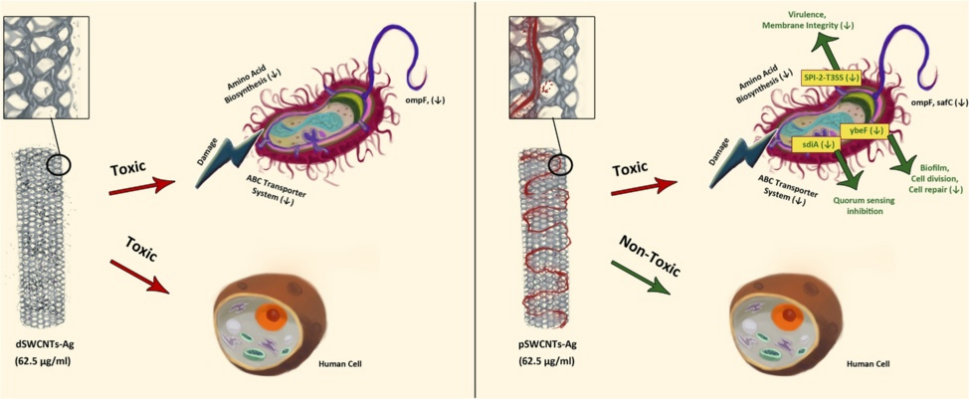
**Schematic presentation of mechanism of action of SWCNTS-Ag and pSWCNTs-Ag.** Our results demonstrated that SWCNTs-Ag down-regulate some of the genes associated with metabolism and outer membrane integrity, however they are still toxic to human cells at their bactericidal concentration (62.5 μg/mL). On the other hand, pegylation of SWCNTs-Ag (pSWCNTs-Ag) did not affect their antibacterial activity (62.5 μg/mL), but reduced their toxicity to human cells. In addition, pSWCNTs down-regulated the expression of genes associated with quorum sensing, biofilm formation and virulence in *Salmonella*.

In conclusion, our results demonstrate that pSWCNTs-Ag are non-toxic to human cells at their bactericidal concentration, and down-regulate the genes associated with quorum sensing and virulence mechanisms in *Salmonella* (Figure 12). Although our EM results show that exposure to SWCNTs and pSWCNTs-Ag produce relatively similar morphological changes in bacteria, qRT-PCR findings clearly underline the differences in gene regulation mechanisms, indicating that pSWCNTs-Ag are more efficient than plain SWCNTs-Ag. Intracellular survival of *Salmonella* and their quorum sensing mechanisms are very important for their pathogenesis [43,44,46]. Treatment of *Salmonella* with pSWCNTs-Ag interrupts these two important mechanisms and thus has an additional advantage over plain SWCNTs-Ag as far as the antibacterial mechanism is concerned. These results show promise towards developing resistance free and safe antimicrobials for the effective treatment of foodborne infections.

## Methods

### Preparation of SWCNTs-Ag solutions

SWCNTs-Ag, with purity greater than 95%, were purchased from NanoLab, Inc. Waltham, MA, USA. The SWCNTs-Ag were produced by catalytic chemical vapor deposition, and had an outer diameter of 1–5 nm and a length of 1–2 μm. SWCNTs-Ag were dispersed in 1 mL of specifically formulated dispersant- NanoSperse AQ® from NanoLab Inc. Briefly, 1 mg of SWCNTs-Ag were suspended in 1 mL of NanoSperse AQ® dispersant solution (according to manufacturer’s instructions) and the suspensions were immediately sonicated for 1-2 h, and shaken for 30 min to obtain the SWCNTs-Ag dispersion (1 mg/mL).

### Functionalization of SWCNTs-Ag using phospholipid-poly(ethylene glycol) (PL-PEG)

The SWCNTs-Ag were non-covalently functionalized using phospholipid-poly(ethylene glycol) PL-PEG5000-Amine (NOF Corporation, White Plains, NY, USA) as described previously [27]. Briefly, hydrophobic SWCNTs-Ag were mixed with water solutions of the amphiphilic polymer PL-PEG5000-Amine in a ratio of 1:5, sonicated for 60 min at room temperature (~22°C) and centrifuged for 6 h (24000 × g), at room temperature. After centrifugation, the supernatant solution containing functionalized SWCNTs- Ag was run through an Amicon centrifugal filter (molecular weight cutoff of 100 kDa Millipore, Billerica, MA, USA) and centrifuged for 10 min (4,000 × g) at room temperature and the sediment was discarded. The functionalized SWCNTs were washed 5–6 times with water to remove excess PL-PEG molecules. Functionalized SWCNTs-Ag were solubilized in sterile nuclease-free water.

### Determination of SWCNTs-Ag concentration in pSWCNTs-Ag solution

To measure the concentration of SWCNTs-Ag in pSWCNTs-Ag solution, the dry weight of the solution was calculated after vacuum drying of the suspension. Simultaneously, the PL-PEG concentration in a solution was determined using an inorganic phosphate assay following acid hydrolysis [47]. Once the concentration of PL-PEG was known, the concentration of SWCNTs-Ag was calculated by subtracting the concentration of PL-PEG from the total dry weight of pSWCNTs-Ag.

### Characterization of SWCNTs-Ag and pSWCNTs-Ag

Both the nanocomposite formulations were characterized using zeta potential and fourier transform infrared spectroscopy (FT-IR) analysis [48].

### Determination of zeta potential

The zeta potential of SWCNTs-Ag and pSWCNTs-Ag was measured using a Zetasizer (Nano-ZS; Malvern Instruments Ltd, Malvern, UK). The samples were diluted in distilled water to 1/10 (v/v), sonicated, and placed in a disposable cuvette for zeta potential measurements. All the measurements were carried out in triplicates for each sample. The values are reported as means of triplicate samples.

### FT-IR spectroscopy

FTIR spectra were recorded for SWCNTs-Ag, pSWCNTs-Ag and PL-PEG5000-amine in attenuated total reflectance (ATR) mode using an infrared (IR) spectrophotometer (Nicolet 380 FT-IR; Thermo Fisher Scientific). The spectra were obtained with 64 scans per sample, ranging from 400 to 4000 cm^−1^ and a resolution of 4 cm^−1^. The sample chamber was purged with dry N2 gas.

### Evaluation of toxicity to eukaryotic cells

#### *In vitro* cell toxicity assay

The cell toxicity to SWCNTs-Ag and pSWCNTs-Ag was determined using Cell Titer 96® Non-Radioactive cell proliferation kits (Promega, Madison, WI). A549 (human lung carcinoma), Hep2 (hepatocellular carcinoma) cells and J774 (murine macrophages) cell lines were used for the cytotoxicity assay. The assay is a colorimetric assay based on the reduction of the tetrazolium dye MTT [3-(4,5-dimethylthiazol-2yl)-2,5-diphenyltetrazolium bromide). As per the manufacturer’s protocol, 1 × 10^4^ cells/well in 100 μL of minimum essential medium-10 (MEM-10, Gibco, Life technologies, Grand Island, NY) were seeded into a 96-well plate. After overnight incubation at 37°C in 5% CO_2_ humidified atmosphere, the media from the 96-well plates were replaced with the MEM-10 media containing 62.5, 31.25, 15.12 or 7.8 μg/mL of either SWCNTs-Ag or pSWCNTs-Ag. The treated cells were further incubated at 37°C under 5% CO_2_ for 24 and 48 h. At the end of the each incubation period, 15 μL of MTT dye were added into each well and the plate was allowed to incubate again for the next 4 h, in darkness. The reaction was then stopped using 100 μL of the stop solution. The absorbance of the plate was measured at 570 nm using a TECAN Sunrise™ enzyme-linked immunosorbent assay (ELISA) plate reader (Tecan US, Inc., Morrisville, NC, USA). Non-treated cells, in growth media, were used as a control.

### DNA fragmentation assay

The DNA fragmentation assay was performed as described elsewhere [49]. Briefly, A549 cells were treated with SWCNTs-Ag and pSWCNTs-Ag at concentrations of 31.25 and 15.6 μg/mL for 48 h, trypsinized and re-suspendeded in 0.5 mL of lysis buffer [10 mM Tris–HCl buffer (pH 8.0) containing 2% (v/v) Triton-X100, 0.5 mM EDTA)], treated with RNAse (10 mg/mL) at 37°C for 2 h and; digested with 200 μg/mL proteinase K for 4 h at 50°C. Upon digestion, DNA was extracted with phenol/chloroform/Iso-amyl alchohol (25:24:1, v/v), and precipitated with 70% ethanol at −20°C overnight. Electrophoresis of the purified DNA was carried out using 1.5% agarose gels containing ethidium bromide and visualized using ultraviolet transillumination.

### Bactericidal experiments

*Salmonella enterica* serovar Typhimurium (ATCC® 13311™), *Escherichia coli* (ATCC® 25922™), *Staphylococcus aureus* (ATCC® 9144™) and *Streptococcus pyogenes* (ATCC® 8135™) purchased from American Type Culture Collection (ATCC, VA USA), were used for the bacterial experiments. Bacteria were grown at 37°C in Luria-Bertani (LB) broth (Difco, Sparks, MD, USA) with continuous shaking until the optical density (OD) was 0.6–0.8 (at 600 nm). Bactericidal activity of SWCNTs-Ag and pSWCNTs-Ag was investigated using the parameters such as minimum inhibitory concentrations (MIC) and the Kirby-Bauer disk diffusion assay against all four pathogens. Further detailed evaluation of the antibacterial effects on *Salmonella* Typhimurium was done by growth curve analysis, and live/dead staining. The morphological changes that occurred in *Salmonella* by treatment with SWCNTs-Ag and pSWCNTs-Ag were examined by scanning electron microscopy (SEM) and transmission electron microscopy (TEM). Gene expression studies were carried out using the quantitative real time reverse transcriptase polymerase chain reaction (qRT-PCR).

### Determination of MIC

The MIC values of SWCNTs-Ag and pSWCNTs-Ag were evaluated in quadruplet wells of sterile 96-well microtiter plates using the broth microdilution assay with or without using the redox reagent resazurin, following a previously described broth microdilution procedure [50,51]. Briefly, early log phase suspensions of the bacteria (1 × 10^5^ cfu/mL) were exposed to doubling concentrations of SWCNTs-Ag and pSWCNTs-Ag starting at 1.9 μg/mL in the presence or absence of resazurin. Two-fold serial dilutions of pSWCNTs-Ag were performed in sterile nuclease free water, whereas the NanoSperse AQ dispersant solution was used as a diluent for the plain SWCNTs-Ag. All plates were sealed lightly (with ventilation) and then incubated at 37°C for 24 h. Each plate consisted of 8 dilutions of the SWCNTs-Ag dispersions/ solutions, 1 negative control (no SWCNTs-Ag or no bacterial culture), and 1 positive control (only bacterial culture without SWCNTs-Ag). After incubation, the MIC was determined by the turbidity of the culture media in the wells. The concentration of the first well without turbidity was considered as the minimum inhibitory concentration. The inhibition of bacterial growth was determined by measuring absorbance at 600 nm with a TECAN Sunrise™ enzyme-linked immunosorbent assay (ELISA) plate reader (Tecan US, Inc Morrisville, NC, USA). While testing plates where resazurin dye was added, MIC values were identified at 24 h as the lowest concentration of antimicrobial agent with color shift from blue to red. All experiments were repeated at least three times.

### Kirby–Bauer disc diffusion assay

The antibacterial activity of SWCNTs-Ag and pSWCNTs-Ag at their MICs such as 62.5 & 31.25 μg/mL was further tested using the Kirby–Bauer disc diffusion assay against all four pathogens as described earlier [52]. Bacterial suspensions of each bacterial strain (10^8^ cfu/mL) were swabbed on the surface of Mueller–Hinton agar plates and filter paper discs (Fisher Scientific, MO) containing 62.5 & 31.25 μg of SWCNTs-Ag and pSWCNTs-Ag, were placed on the plate. A broad spectrum combination of amoxicillin and clavulanic acid (30 μg) was used as a positive control (BD, BBL™, USA). Plates were incubated at 37°C overnight and the diameters of the clear zones around the discs, called ‘zones of inhibition’, were recorded.

### Quantitative growth analysis of *Salmonella*

*Salmonella* growth was quantified sequentially at time points of 0, 4, 8, 16 and 24 h post-exposure to SWCNTs-Ag or pSWCNTs-Ag. Twenty milliliters of cultures containing 1 × 10^5^ cfu/mL of bacteria were exposed to 50, 25, 12.5 and 6.25 μg/mL of SWCNTs-Ag, or 125, 62.5, 31.2 and 15.7 μg/mL of pSWCNTs-Ag (these concentrations were selected based on the MICs of pSWCNTs-Ag and plain SWCNTs-Ag). The cultures were incubated at 37°C with shaking at 250 rpm and the optical density at 600 nm (OD600) was determined at 0, 4, 8, 16 and 24 h. The graph was plotted as O.D vs. time point on the Y and X axis, respectively. Similarly, at each time point, 1 mL aliquots of bacterial culture were collected, subjected to serial 10-fold dilution in sterile LB broth, and each dilution was then spread on PCA to determine the cfu/mL. Each sample was analyzed in triplicate and the analysis was repeated at least three times. The average cfu/mL value of each sample was then reported as described above.

### Live/dead staining of bacteria

The viability of bacteria was examined by live/dead staining using the Baclight bacterial viability kit (L13152, Molecular probes, USA) according to manufacturer’s instructions. Briefly, 1 × 10^5^ cfu/mL bacteria were treated with 12.5 μg/mL of SWCNTs-Ag and pSWCNTs-Ag for 16 h in a shaking incubator (250 rpm) at 37°C. Post-treatment, the bacterial cells were harvested, washed with 0.85% sodium chloride (NaCl, Fischer Thermo scientific, New Jersey, USA), incubated further in 0.85% NaCl for 1 h and finally re-suspended in 200 μl of 0.85%Nacl. Bacterial samples were then incubated in the dark for 30–45 min with an equal amount of 2× stock solution of the LIVE/DEAD BacLight staining reagent containing a final concentration of 6 μM of the SYTO 9 dye and 30 μM of propidium iodide. The stained bacterial suspensions were trapped (5 μl) between a slide and an 18 mm square coverslip and the images were captured by Nikon Eclipse TE200 microscope (Nikon, Melville, NY, USA) using FITC-HYQ (Ex 450–500) and TRITC HYQ (Ex 530–550) filters. Viable cells had green fluorescence, while non-viable cells had red fluorescence. All the experiments were carried out in triplicate sets and the treated samples were compared with non-treated controls. The live and dead cells (of at least three images of treated and non-treated bacteria) were counted manually using NIS-Elements AR 3.1 microscope imaging software and the live/dead ratio for each sample was calculated.

### Electron microscopy

Scanning electron microscopy (SEM, Zeiss EVO 50, Carl Zeiss Meditec, Oberkochen, Germany) and transmission electron microscopy (TEM, Zeiss EM 10C 10CR, Carl Zeiss Meditec, Oberkochen, Germany) were used to examine the morphology and size of SWCNTs-Ag and pSWCNTs-Ag. Additionally, SEM and TEM were used to observe the morphological features of *Salmonella* Typhimurium treated with SWCNTs-Ag and pSWCNTs-Ag. The non-treated bacterial cells were used as a control. Similarly, TEM analysis was performed to investigate the cytotoxicity of the Ag nanocomposites in A549 cells compared to non-treated cells. Samples of SWCNTs-Ag and pSWCNTs-Ag for SEM were prepared as described previously with minor modifications [8]. Briefly, the samples were sonicated, diluted 10 times and placed on a SEM stub with a conductive silver paint and sputter-coated with gold/palladium using a sputter coat device (EMS 550×, Carl Zeiss Meditec, Oberkochen, Germany). For bacterial sample preparation, cells (1 × 10^5^ cfu/mL) were treated with 12.5 μg/mL of SWCNTs-Ag and pSWCNTs-Ag separately for 16 h in a shaking incubator (250 rpm) at 37°C. For eukaryotic sample preparation, A549 cells were treated with 31.25 & 15.6 μg/mL of SWCNTs-Ag and pSWCNTs-Ag separately for 48 h and incubated at 37°C in 5% CO_2_ humidified atmosphere. Post treatment, the bacterial cells were harvested by centrifugation (13000 × **g** for 10 min), fixed in 2.5% glutaraldehyde and 1% formaldehyde overnight, followed by fixation in 1% aqueous osmium tetroxide for 1 h and then subjected to serial dehydration in increasing percentages of ethanol (30, 50, 70, 80, 90, 95 and 100%). After the final dehydration, step, 5 μl of each sample was placed on the SEM stub, air dried and sputter coated with a gold-palladium alloy for subsequent observation by SEM. For TEM, the 10 fold diluted samples of SWCNTs-Ag and pSWCNTs-Ag were dispersed onto the carbon-coated copper grids (200 mesh). The bacterial and A549 cells samples were passed through propylene oxide, polymerized in Embed 812 resin, followed by ultrathin gold colored sectioning on to copper grids and observation by TEM.

### Molecular studies using qRT-PCR

The mRNA levels of several metabolically essential genes and virulence factor-associated genes of *Salmonella* Typhimurium were investigated using qRT-PCR. The primer sets and the function of each gene are described in Table 2. The bacteria (1 × 10^5^ cfu/mL) were treated with 12.5 μg/mL of SWCNTs-Ag and pSWCNTs-Ag for 16 h in a shaking incubator (250 rpm) at 37°C, followed by total RNA extraction using RNeasy Mini kit (Qiagen, Germany). The RNA was quantified by using a NanoVue Plus spectrophotometer at 260 nm/280 nm (GE Healthcare Life Sciences, Pittsburg, PA), and cDNA synthesis was carried out in a 40 μl reaction volume using the Applied Biosystems High Capacity cDNA Reverse Transcriptase Kit (Life Technologies, Grand Island, NY). The expression of metabolically essential genes such as genes associated with ABC transporter system (*dppA*, *artP* and *livJ*), amino acid biosynthesis (*trp* and *argC*) and DNA transcription (*sdiA*, *fliH* and *ybeF*); virulence factor associated genes such as *Salmonella* pathogenicity island-2 (SPI) (*ssaH*, *sseA*, *sseB* and *sseG*), SPI-3 (*cigR*), SPI-6 (*safC*), outer membrane protein (*ompF*) and invasion (*ychP*) genes were quantified by qRT-PCR using the SYBR® Select Mastermix (Life Technologies, Grand Island, NY) according to the manufacturer’s instructions. DNA was amplified and quantified in the Applied Biosystems® ViiA™ 7 real-time PCR system (Life Technologies). PCR conditions consisted of an initial denaturation at 95°C for 2mins, followed by 40 cycles of 95°C for 5 s, 56°C for 25 s and 72°C for 30s. Amplification efficiency of each primer set with respect to the endogenous control gene (*16srRNA*) was in between 95-98% (data not shown). The data obtained from three-independent experiments were used to analyze the relative gene expression compared to non-treated samples by the 2 − ΔΔCt method [53].

**Table 2:** **Primers used in this study**

**Gene**	**Forward (5′-3′)**	**Reverse (5′-3′)**	**Function**
*sseA*	GGGCTAAGGTGAGTCAACA	TGAAGAATACTCTCTGTCTCTCTG	Virulence (SPI-2 associated)
*sseB*	CATCTTATGGGGAAGTCAAAACC	GATAAGTCATCCTGGCTCCC	Virulence (SPI-2 associated)
*ssah*	TGATTTCCCAGGTACATGCGATG	GCCAACAATAATGCCAGACATACC	Virulence (SPI-2 associated)
*sseG*	GCCGATCCCAGTGTGTTATT	GCATTTGGGCTAACAGGTTTC	Virulence (SPI-2 associated)
*ychP*	GCGACCTTAGAACAGCGAAT	CCAAAGTACTGCTCCAGACTAAC	Invasion
*cigR*	CCCACATCAGAAGGGCAATATC	GCCATTACCATTTCCCGGATT	Inner membrane protein
*safC*	ATGGTAGCGCCATTCCTTTC	CCGCCAAACCAGTGAGATAAA	Outer membrane protein
*ompF*	CGTTGGCGCGAAATATGATG	GTTTGCCATTTCCACGGTATC	Outer membrane protein F
*ybeF*	TCCCGATCCGCTGTTTATTC	GCTTATCATAGCTGCCCGTAA	DNA transcription activator
*FliH*	TCGTCTGTCTCAGGTGGAA	CTTGCCACGGCAATTCATTAG	DNA transcription activator
*sdiA*	GCCCAGCGTTTCGGATTA	GTAAACGAGGAGCAGCGTAAA	DNA transcription, quorum sensing
*trpA*	CAGCCATTGTCAAGATTATCG	CTGAGACAAAGGACCTGAG	Tryptophan biosynthesis
*argC*	CAGTTTCTGTGAAGTGAGTTT	CAGATGGACGGCGATTTC	Arginine biosynthesis
*dppA*	ATCAAAGCCGTTTATCAG	TTAATATCGTCGTTGTAGC	ABC transporter system
*artP*	GTCTTTCAGCAATATAATCTTTGG	TCTTTTGTCAGACCCAGTA	ABC transporter system
*livJ*	CAACGGCGGCAAAGTATA	CGTACTGCTGCTTATCGT	ABC transporter system
*16 s rRNA*	CAGAAGAAGCACCGGCTAAC	AATGCAGTTCCCAGGTTGAG	Endogenous control

### Statistical analyses

All data are expressed as the mean ± standard deviation (SD) unless otherwise specified. Analyses were performed using the GraphPad Prism Version 4 software (GraphPad Software, Inc., La Jolla, CA). Statistical differences for growth curves were evaluated by using two way ANOVA. Fold change expression of genes were analyzed by the Student’s t test. Differences were considered to be statistically significant when the P-values were ≤0.05 or 0.01.

## Additional file

Additional file 1:
**Antibacterial activity of SWCNTs-Ag and pSWCNTs-Ag against gram-positive and gram- negative bacteria.**

## Abbreviations

AgNPs: Silver nanoparticles
AgCNTs: Silver coated CNTs
ANOVA: Analysis of variance
ATR: Attenuated total reflectance
CNTs: Carbon nanotubes
ELISA: Enzyme-linked immunosorbent assay
EM: Electron microscopy
FT-IR: Fourier transform infrared spectroscopy
IR: Infrared
MIC: Minimum inhibitory concentrations
MTT: 3-(4,5-dimethylthiazol-2yl)-2,5-diphenyltetrazolium bromide
NP: Nanoparticles
OD: Optical density
PEG: Polyethylene glycol
PL-PEG: Phospholipid-poly(ethylene glycol)
SWCNTs-Ag: Silver coated single walled carbon nanotubes
pSWCNTs-Ag: Pegylated SWCNTS-Ag
qRT-PCR: Quantitative reverse transcriptase polymerase chain reaction
SEM: Scanning electron microscopy
TEM: Transmission electron microscopy

## References

[1] Durso LM, Cook KL (2014). Impacts of antibiotic use in agriculture: what are the benefits and risks?. Curr Opin Microbiol.

[2] Pelgrift RY, Friedman AJ (2013). Nanotechnology as a therapeutic tool to combat microbial resistance. Adv Drug Deliv Rev.

[3] Chatterjee S, Bandyopadhyay A, Sarkar K (2011). Effect of iron oxide and gold nanoparticles on bacterial growth leading towards biological application. J Nanobiotechnol.

[4] Huh AJ, Kwon YJ (2011). “Nanoantibiotics”: a new paradigm for treating infectious diseases using nanomaterials in the antibiotics resistant era. J Control Release.

[5] Dallas P, Sharma VK, Zboril R (2011). Silver polymeric nanocomposites as advanced antimicrobial agents: classification, synthetic paths, applications, and perspectives. Adv Colloid Interface Sci.

[6] Guzman M, Dille J, Godet S (2012). Synthesis and antibacterial activity of silver nanoparticles against gram-positive and gram-negative bacteria. Nanomedicine.

[7] Kurek A, Grudniak AM, Kraczkiewicz-Dowjat A, Wolska KI (2011). New antibacterial therapeutics and strategies. Pol J Microbiol.

[8] Rangari VK, Mohammad GM, Jeelani S, Hundley A, Vig K, Singh SR (2010). Synthesis of Ag/CNT hybrid nanoparticles and fabrication of their nylon-6 polymer nanocomposite fibers for antimicrobial applications. Nanotechnology.

[9] Su R, Jin Y, Liu Y, Tong M, Kim H (2013). Bactericidal activity of Ag-doped multi-walled carbon nanotubes and the effects of extracellular polymeric substances and natural organic matter. Colloids Surf B Biointerfaces.

[10] Brahmachari S, Duttaa S, Das PK (2014). Biotinylated amphiphile-single walled carbon nanotube conjugate for target-specific delivery to cancer cells. J Mater Chem B.

[11] Jung JH, Hwang GB, Lee JE, Bae GN (2011). Preparation of airborne Ag/CNT hybrid nanoparticles using an aerosol process and their application to antimicrobial air filtration. Langmuir.

[12] Li Z, Fan L, Zhang T, Li K (2011). Facile synthesis of Ag nanoparticles supported on MWCNTs with favorable stability and their bactericidal properties. J Hazard Mater.

[13] Brahmachari S, Mandal SK, Das PK (2014). Fabrication of SWCNT-Ag nanoparticle hybrid included self-assemblies for antibacterial applications. PLoS One.

[14] Brahmachari S, Das D, Shome A, Das PK (2011). Single-walled nanotube/amphiphile hybrids for efficacious protein delivery: rational modification of dispersing agents. Angew Chem Int Ed Engl.

[15] Ahamed M, Alsalhi MS, Siddiqui MK (2010). Silver nanoparticle applications and human health. Clin Chim Acta.

[16] Faedmaleki F, Shirazi F, Salarian AA, Ahmadi Ashtiani H, Rastegar H (2014). Toxicity effect of silver nanoparticles on mice liver primary cell culture and hepg2 cell line. Iran J Pharm Res.

[17] Kovvuru P, Mancilla PE, Shirode AB, Murray TM, Begley TJ, Reliene R. Oral ingestion of silver nanoparticles induces genomic instability and DNA damage in multiple tissues. Nanotoxicology. 2014. Epub ahead of print. 10.3109/17435390.2014.902520 24713076

[18] Park EJ, Yi J, Kim Y, Choi K, Park K (2010). Silver nanoparticles induce cytotoxicity by a Trojan-horse type mechanism. Toxicol In Vitro.

[19] Park S, Lee YK, Jung M, Kim KH, Chung N, Ahn EK (2007). Cellular toxicity of various inhalable metal nanoparticles on human alveolar epithelial cells. Inhal Toxicol.

[20] Jain S, Singh SR, Pillai S (2012). Toxicity issues related to biomedical applications of carbon nanotubes. J Nanomed Nanotechol.

[21] Vardharajula S, Ali SZ, Tiwari PM, Eroglu E, Vig K, Dennis VA (2012). Functionalized carbon nanotubes: biomedical applications. Int J Nanomedicine.

[22] Dumortier H, Lacotte S, Pastorin G, Marega R, Wu W, Bonifazi D (2006). Functionalized carbon nanotubes are non-cytotoxic and preserve the functionality of primary immune cells. Nano Lett.

[23] Haberl N, Hirn S, Wenk A, Diendorf J, Epple M, Johnston BD (2013). Cytotoxic and proinflammatory effects of PVP-coated silver nanoparticles after intratracheal instillation in rats. Beilstein J Nanotechnol.

[24] Schipper ML, Nakayama-Ratchford N, Davis CR, Kam NW, Chu P, Liu Z (2008). A pilot toxicology study of single-walled carbon nanotubes in a small sample of mice. Nat Nanotechnol.

[25] Tejamaya M, Romer I, Merrifield RC, Lead JR (2012). Stability of citrate, PVP, and PEG coated silver nanoparticles in ecotoxicology media. Environ Sci Technol.

[26] Thorley AJ, Tetley TD (2013). New perspectives in nanomedicine. Pharmacol Ther.

[27] Liu Z, Tabakman SM, Chen Z, Dai H (2009). Preparation of carbon nanotube bioconjugates for biomedical applications. Nat Protoc.

[28] Chou CC, Hsiao HY, Hong QS, Chen CH, Peng YW, Chen HW (2008). Single-walled carbon nanotubes can induce pulmonary injury in mouse model. Nano Lett.

[29] Chen M, Yang Z, Wu H, Pan X, Xie X, Wu C (2011). Antimicrobial activity and the mechanism of silver nanoparticle thermosensitive gel. Int J Nanomedicine.

[30] Chen H, Wang B, Gao D, Guan M, Zheng L, Ouyang H (2013). Broad-spectrum antibacterial activity of carbon nanotubes to human gut bacteria. Small.

[31] Kang S, Herzberg M, Rodrigues DF, Elimelech M (2008). Antibacterial effects of carbon nanotubes: size does matter!. Langmuir.

[32] Szostak MP, Hensel A, Eko FO, Klein R, Auer T, Mader H (1996). Bacterial ghosts: non-living candidate vaccines. J Biotechnol.

[33] Stukalov O, Korenevsky A, Beveridge TJ, Dutcher JR (2008). Use of atomic force microscopy and transmission electron microscopy for correlative studies of bacterial capsules. Appl Environ Microbiol.

[34] Pawley J (1997). The development of field-emission scanning electron microscopy for imaging biological surfaces. Scanning.

[35] Blair JM, Richmond GE, Bailey AM, Ivens A, Piddock LJ (2013). Choice of bacterial growth medium alters the transcriptome and phenotype of *Salmonella enterica* Serovar Typhimurium. PLoS One.

[36] Gong H, Vu GP, Bai Y, Chan E, Wu R, Yang E (2011). A *Salmonella* small non-coding RNA facilitates bacterial invasion and intracellular replication by modulating the expression of virulence factors. PLoS Pathog.

[37] Davidson AL, Dassa E, Orelle C, Chen J (2008). Structure, function, and evolution of bacterial ATP-binding cassette systems. Microbiol Mol Biol Rev.

[38] Giaouris E, Samoilis G, Chorianopoulos N, Ercolini D, Nychas GJ (2013). Differential protein expression patterns between planktonic and biofilm cells of *Salmonella enterica* serovar Enteritidis PT4 on stainless steel surface. Int J Food Microbiol.

[39] Wissenbach U, Six S, Bongaerts J, Ternes D, Steinwachs S, Unden G (1995). A third periplasmic transport system for L-arginine in *Escherichia coli*: molecular characterization of the artPIQMJ genes, arginine binding and transport. Mol Microbiol.

[40] Matsubara K, Ohnishi K, Sadanari H, Yamada R, Fukuda S (2000). A portion of the nucleotide sequence corresponding to the N-terminal coding region of *livJ* is essential for its transcriptional regulation. Biochim Biophys Acta.

[41] Guyard C, Raffel SJ, Schrumpf ME, Dahlstrom E, Sturdevant D, Ricklefs SM (2013). Periplasmic flagellar export apparatus protein, FliH, is involved in post-transcriptional regulation of *FlaB*, motility and virulence of the relapsing fever spirochete *Borrelia hermsii*. PLoS One.

[42] Espinosa E, Casadesus J (2014). Regulation of S*almonella enterica* pathogenicity island 1 (SPI-1) by the LysR-type regulator LeuO. Mol Microbiol.

[43] Smith JL, Fratamico PM, Yan X (2011). Eavesdropping by bacteria: the role of SdiA in *Escherichia coli* and *Salmonella enterica* serovar Typhimurium quorum sensing. Foodborne Pathog Dis.

[44] Marcus SL, Brumell JH, Pfeifer CG, Finlay BB (2000). *Salmonella* pathogenicity islands: big virulence in small packages. Microbes Infect.

[45] Zurawski DV, Stein MA (2004). The SPI2-encoded SseA chaperone has discrete domains required for SseB stabilization and export, and binds within the C-terminus of SseB and SseD. Microbiology.

[46] Gnanendra S, Mohamed S, Natarajan J (2013). Identification of potent inhibitors for *Salmonella* typhimurium quorum sensing via virtual screening and pharmacophore modeling. Comb Chem High Throughput Screen.

[47] Bartlett GR (1959). Phosphorus assay in column chromatography. J Biol Chem.

[48] Eroglu E, Tiwari PM, Waffo AB, Miller ME, Vig K, Dennis VA (2013). A nonviral pHEMA + chitosan nanosphere-mediated high-efficiency gene delivery system. Int J Nanomedicine.

[49] Seo JS, Moon MH, Jeong JK, Seol JW, Lee YJ, Park BH (2012). SIRT1, a histone deacetylase, regulates prion protein-induced neuronal cell death. Neurobiol Aging.

[50] Palomino JC, Martin A, Camacho M, Guerra H, Swings J, Portaels F (2002). Resazurin microtiter assay plate: simple and inexpensive method for detection of drug resistance in *Mycobacterium tuberculosis*. Antimicrob Agents Chemother.

[51] Soehnlen MK, Kunze ME, Karunathilake KE, Henwood BM, Kariyawasam S, Wolfgang DR (2011). *In vitro* antimicrobial inhibition of *Mycoplasma bovis* isolates submitted to the Pennsylvania Animal Diagnostic Laboratory using flow cytometry and a broth microdilution method. J Vet Diagn Invest.

[52] Bauer AW, Kirby WM, Sherris JC, Turck M (1966). Antibiotic susceptibility testing by a standardized single disk method. Am J Clin Pathol.

[53] Schmittgen TD, Livak KJ (2008). Analyzing real-time PCR data by the comparative C(T) method. Nat Protoc.

