# Supplementary material for: Novel pegylated silver coated carbon nanotubes kill Salmonella but they are non-toxic to eukaryotic cells
Source: J Nanobiotechnology. 2015 Mar 22;13:23. doi: 10.1186/s12951-015-0085-5 (PMC4377206; doi:10.1186/s12951-015-0085-5)
Supplement: Additional file 1: — Antibacterial activity of SWCNTs-Ag and pSWCNTs-Ag against gram-positive and gram- negative bacteria. [file 12951_2015_85_MOESM1_ESM.doc]

**Additional File 1**

**Novel pegylated silver coated carbon nanotubes kill *Salmonella* but they are non-toxic to eukaryotic cells**

Atul A. Chaudhari^1^, Shanese L. Jasper^1^, Ejovwoke Dosunmu^1^, Michael E. Miller^2^, Robert D. Arnold^3^, Shree R. Singh^1^, Shreekumar Pillai^1^*

^1^Center for Nanobiotechnology Research, Alabama State University, Montgomery, AL, USA

^2^ Research Instrumentation Facility, Auburn University, AL, USA

^3^ Department of Drug Discovery and Development, Auburn University, AL, USA

Corresponding Author:

* E-mail: spillai@alasu.edu

**Supplementary information**

**Table S1.** **Diameters of zones of inhibition for four bacterial pathogens measured by the Kirby–Bauer disc diffusion assay.**

| Bacteria | Zone of inhibition (mm) | | | | |
| --- | --- | --- | --- | --- | --- |
|  | SWCNTs-Ag (n=3) | | pSWCNTs-Ag (n=3) | | Amoxicillin-clavulanic acid (n=3) |
|  | 62.5 µg | 31.25 µg | 62.5 µg | 31.25 µg |  |
| *Salmonella* Typhimurium | 16.0 ± 2.6 | 10.0 ± 1.5 | 15.6 ± 1.5 | 11.3 ± 1.5 | 21.0 ± 1.0 |
| *E.coli* | 12.6 ± 1.5 | 11.0 ± 1.7 | 13.0 ± 1.0 | 11.0 ± 1.0 | 16.0 ± 1.0 |
| *Streptococcus pyogenes* | 14.6 ± 2.0 | 10.0 ± 1.0 | 16.0 ± 1.7 | 11.6 ± 1.5 | 19.0 ± 1.0 |
| *Staphylococcus aureus* | 15.6 ± 2.5 | 10.3 ± 1.5 | 15.0 ± 2.6 | 10.3 ± 1.5 | 18.0 ± 1.0 |

**Figures**

**
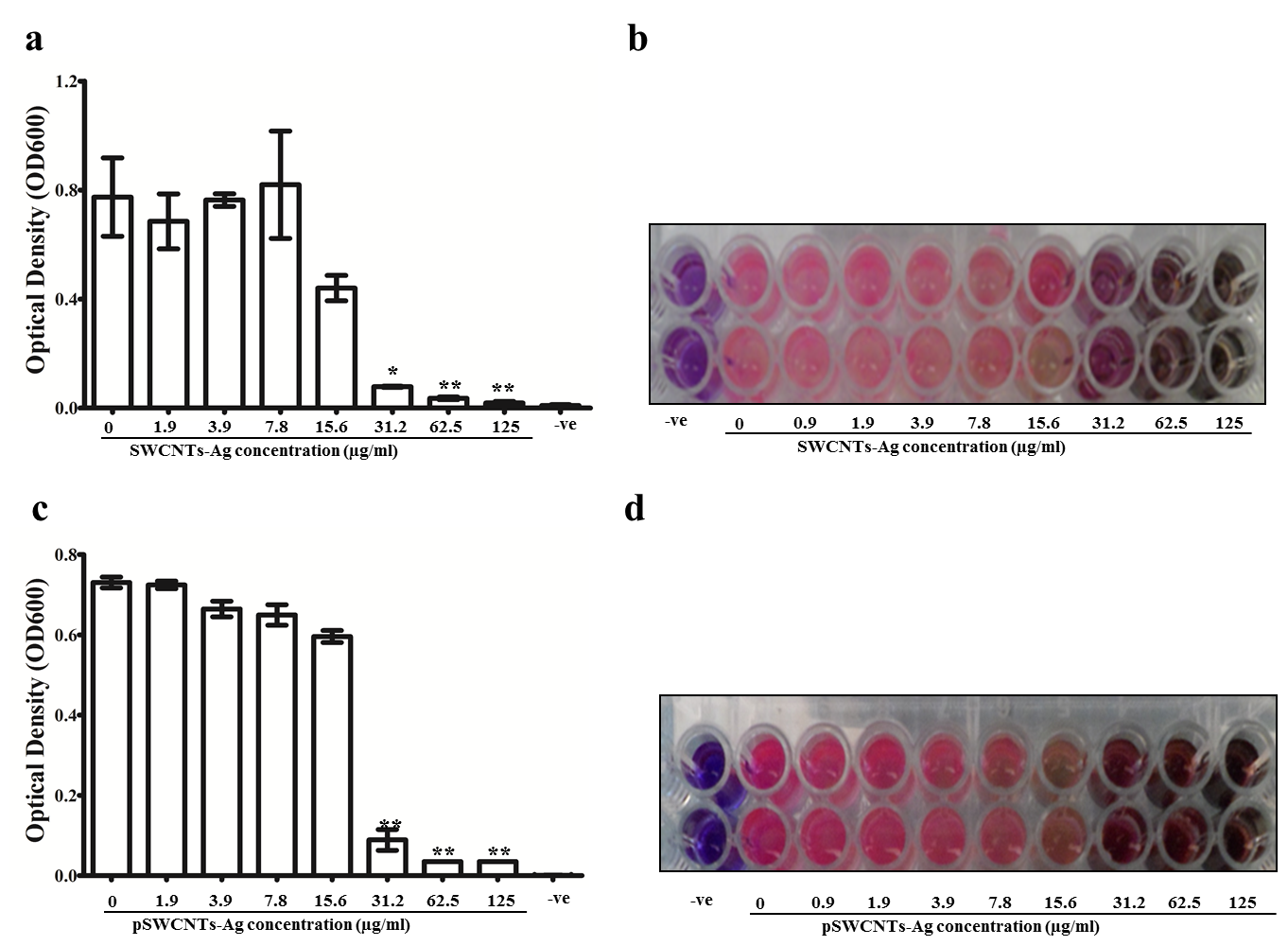
**

**Supplemental Figure S1.** **Evaluation of the minimum inhibitory concentrations (MICs) using the redox resazurin dye-based microtiter broth dilution assay.** 1 X 10^5^ cfu/mL *Staphylococcus aureus* were exposed to doubling concentrations of nanocomposites. (a) SWCNTs-Ag without resazurin. (b) SWCNTs-Ag with resazurin. (c) pSWCNTs-Ag without resazurin. (d) pSWCNTs-Ag with resazurin. All the plates were incubated at 37 °C and the optical density at 600 nm (OD600) was determined after 24 h. All values were considered to be significant if p ≤ 0.05 or 0.01 versus the controls (0 µg/mL of SWCNTs-Ag present with bacterial culture). ** When p ≤ 0.01 indicate highly significant differences. Error bars represent standard deviations determined from at least six replicates.


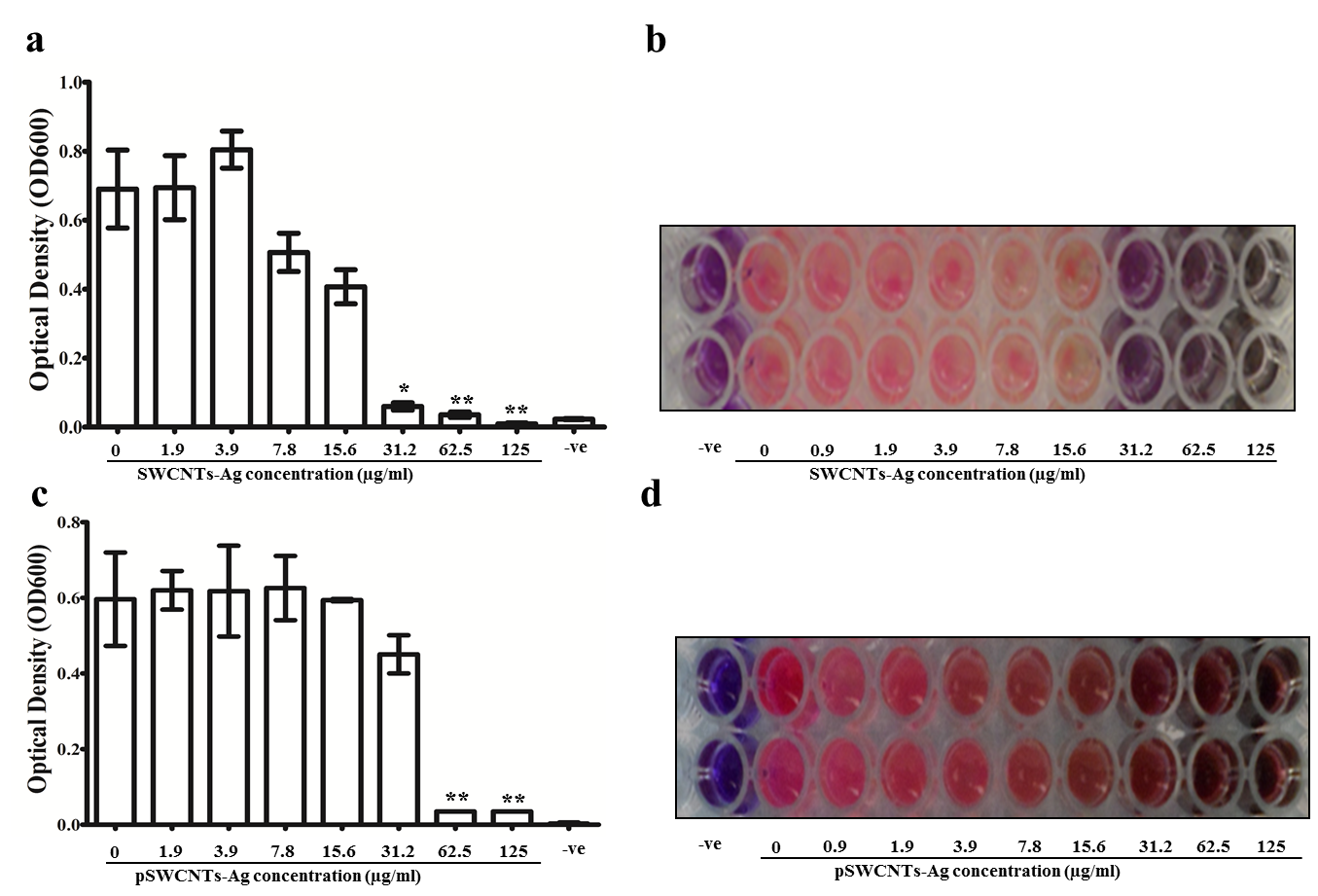


**Supplemental Figure S2.** **MIC against *Streptococcus pyogenes*.** (a) SWCNTs-Ag without resazurin. (b) SWCNTs-Ag with resazurin. (c) pSWCNTs-Ag without resazurin. (d) pSWCNTs-Ag with resazurin. All the plates were incubated at 37 °C and the optical density at 600 nm (OD600) was determined after 24 h. All values were considered to be significant if p ≤ 0.05 or 0.01 versus the controls (0 µg/mL of SWCNTs-Ag present with bacterial culture). ** When p ≤ 0.01 indicate highly significant differences. Error bars represent standard deviations determined from at least six replicates.


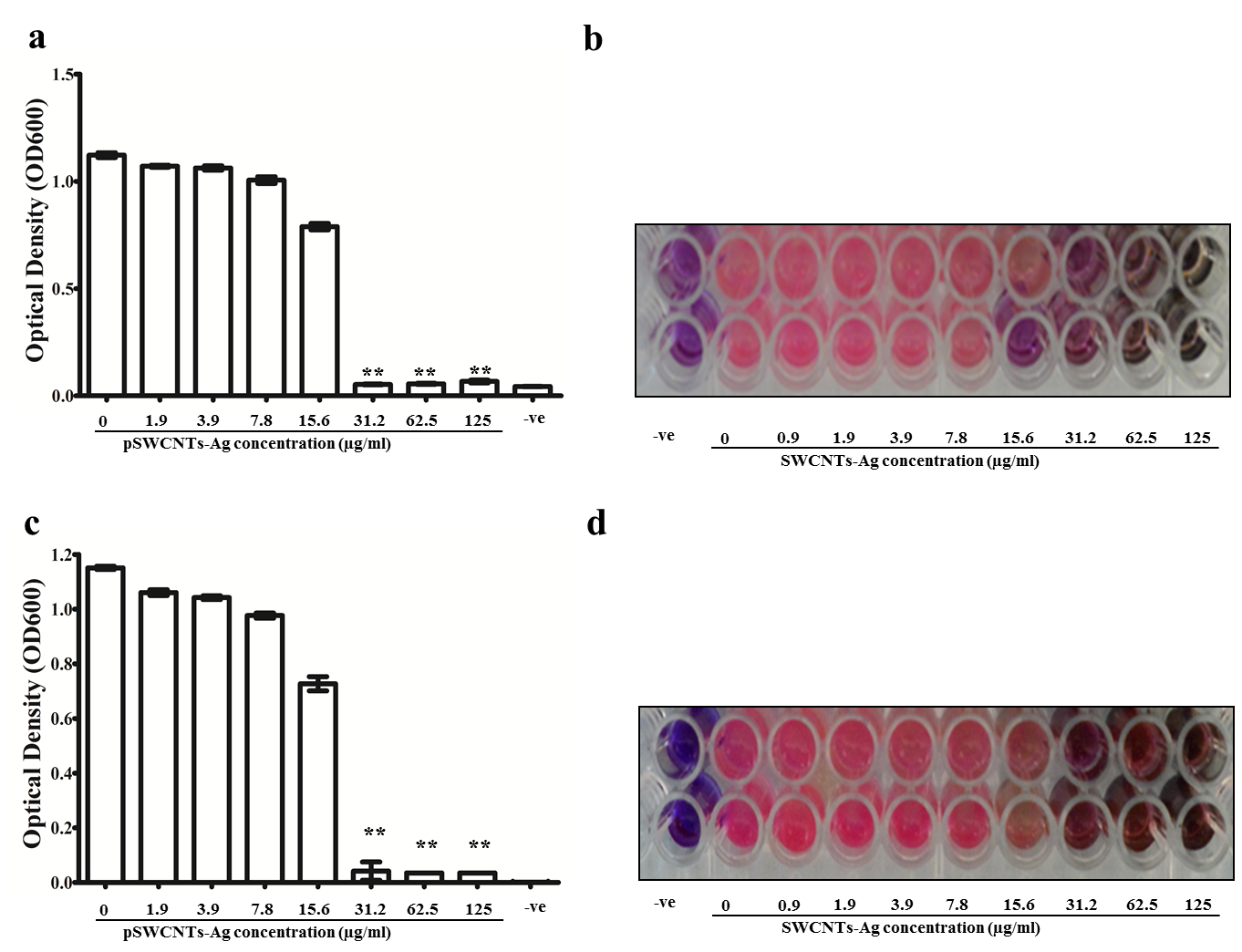


**Supplemental Figure S3.** **MIC against *Escherichia coli*.** (a) SWCNTs-Ag without resazurin. (b) SWCNTs-Ag with resazurin. (c) pSWCNTs-Ag without resazurin. (d) pSWCNTs-Ag with resazurin. All the plates were incubated at 37 °C and the optical density at 600 nm (OD600) was determined after 24 h. All values were considered to be significant if p ≤ 0.05 or 0.01 versus the controls (0 µg/mL of SWCNTs-Ag present with bacterial culture). ** When p ≤ 0.01 indicate highly significant differences. Error bars represent standard deviations determined from at least six replicates.
